# Supplementary material for: Patient acceptance of preventive antibiotic treatment for tuberculosis: a qualitative study in London, UK
Source: BMJ Open. 2026 Oct 23;16(10):e126587. doi: 10.1136/bmjopen-2026-126587 (PMC13711513; doi:10.1136/bmjopen-2026-126587)
Supplement: Supplementary file 1 [file bmjopen-2026-126587.reviewer_comments.pdf]

PEER REVIEW HISTORY

BMJ Open publishes all reviews undertaken for accepted manuscripts. Reviewers are asked to complete a checklist review form and are provided with free text boxes to elaborate on their assessment. These free text comments are reproduced below.

ARTICLE DETAILS

Title (Provisional)

Patient acceptance of preventive antibiotic treatment for tuberculosis: a qualitative study in London, UK

Authors

Antunes, Mélanie; Rose-Key, Rubin; Steward, Ellen; Assayad, Sammy; Noursadeghi, Mahdad; Loría-Rebolledo, Luis Enrique; Crayton, Elisa; Gupta, Rishi

---

VERSION 1 - REVIEW

**Reviewer**	**1**
**Name**	**Xu, Lin**
**Affiliation**	**Yunnan Center for Disease Control and Prevention, Division of tuberculosis control and prevention**
**Date**	**25-Aug-2026**
**COI**	**None**

This study was designed and conducted very well, and it was helpful for public health implications. But there were some points needed to be revised.

1.The manuscript did not provided ethics approval.

2.The study recruited participants from only two London hospitals. But London as a high-migrant-density city with relatively high TB incidence may have different healthcare accessibility compared to other UK regions. The discussion does not sufficiently address this unique characteristics of London. Recommendation: Expand the discussion to elaborate on the scope of generalisability, explicitly cautioning against uncritical application of findings to rural low-incidence areas in the UK or other national contexts.

3.The study mentioned telephone translation services for non-English speakers but did not specify how many participants used this service or discuss the potential impact of translation on data quality. In qualitative research, translation may alter semantic nuances. Recommendation: Supplement the methods with information on the number of participants requiring translation, the languages involved, and a discussion of how translation-related challenges were addressed (e.g., back-translation verification).

4.The study found that knowledge had a mixed influence on treatment decisions, but did not adequately explore how knowledge interacts with other domains (e.g., self-efficacy, perceived risk). Understanding these interactions could inform more effective patient education interventions.Recommendation: Enhance the discussion with an analysis of potential interaction effects between knowledge and other TDF domains, which may provide valuable insights for designing multifaceted interventions.

Reviewer **2**

Name **Arini, Merita**

Affiliation **Universitas Muhammadiyah Yogyakarta, Family Medicine and Public Health**

Date **03-Sep-2026**

COI **None**

Overall, this study is well conducted, well structured, and provides a clear description of the study context, methodology, and findings. One of the strengths of this study is that it captures perspectives from a diverse range of informants, including those who accepted TB preventive therapy, those who declined it, and those who remained undecided. This provides a relatively comprehensive and nuanced understanding of the factors shaping decision-making around TB preventive therapy.

Below are several suggestions that may further strengthen the manuscript:

1. Provide more context on the UK TB prevention and healthcare system. The UK, as a low-TB-burden country, provides an interesting and relatively unique context for this study. However, not all international readers may be familiar with how TB prevention and control programs are organized and delivered in the UK, including the financing and access mechanisms. I suggest adding a brief description of the UK TB prevention and control context in the Introduction, including how TB preventive therapy is offered, delivered, and financed. This would help international readers better understand the context in which the study was conducted and, importantly, allow them to draw broader lessons from the UK experience.

2. Reconsider the terminology “Prospective Cohort Qualitative Study." The term “Prospective Cohort Qualitative Study” feels somewhat unusual and may confuse readers. The term “cohort” is strongly associated with quantitative epidemiological research, in which participants are typically followed to estimate risks or associations using statistical methods. Therefore, I would suggest using a more conventional term, such as "Qualitative Longitudinal Research (QLR)" or "Prospective Qualitative Study," if this accurately reflects the actual study design.

3. Clarify the basis for describing the study as prospective. Related to the previous point, how do the authors justify describing this study as “prospective” if there were no repeated interviews or periodic observations of the participants? In qualitative prospective or longitudinal research, participants are generally followed over time, with qualitative data collected at multiple time points (e.g., through repeated in-depth interviews or observations). The purpose is not necessarily to estimate incidence or risk, but to explore how perceptions, experiences, emotions, social dynamics, or decisions evolve over

time. The authors, therefore, need to clarify what makes the present study prospective. If participants were not followed longitudinally, it may be more appropriate to describe the design as an "exploratory qualitative study" rather than a prospective qualitative study.

4. Reconsider the phrase “mixed influence on treatment acceptance.” The expression “mixed influence on treatment acceptance” feels somewhat inconsistent with the qualitative nature of this study, particularly since it does not seek to establish causal relationships between variables. I suggest using terminology that better reflects the interpretive nature of qualitative findings, for example, “dual considerations in treatment acceptance decisions,” “competing considerations in treatment decision-making,” or similar wording that captures how a particular factor may be perceived in different ways by participants.

5. Further explain the absence of factors mapped to “Skills” and “Reinforcement.” The statement that “No factor was mapped into the domains of ‘Skills’ or ‘Reinforcement’ deserves further explanation and discussion in the Discussion section. This finding is potentially important, but it may be difficult for readers to interpret without first understanding the specific context of TB prevention in the UK and how TB preventive therapy is offered and managed within the healthcare system. At present, readers may be somewhat surprised by this finding. The authors could therefore discuss why these domains were not prominent in participants’ accounts and whether this may be related to the characteristics of TB preventive therapy delivery in a low-burden setting.

6. Strengthen the discussion through comparison with contrasting evidence and different TB-burden settings. The Discussion could be further strengthened by engaging with studies reporting findings that differ from, or even contradict, those observed in the present study. In addition, comparison with evidence from both high-TB-burden and low-TB-burden countries would be valuable. Such comparisons could help demonstrate whether the findings are context-specific or reflect broader patterns in TB preventive therapy decision-making. This would also provide a stronger basis for articulating the novelty and contribution of the present study to the existing literature.

7. Elaborate further on the health-system and health-promotion implications. Finally, the implications of these findings for TB prevention and control in high-income, low-TB-burden settings deserve further elaboration. In particular, the authors could discuss how TB preventive therapy programs should be designed and communicated in such settings, and how health promotion and patient communication strategies may need to differ when TB is perceived as relatively uncommon. This discussion could be strengthened by integrating relevant findings from recent studies and current international guidelines and recommendations on TB prevention, particularly those addressing TB preventive therapy, patient engagement, and health-system approaches in low-burden settings.

VERSION 1 - AUTHOR RESPONSE

Reviewer: 1

This study was designed and conducted very well, and it was helpful for public health implications. But there were some points needed to be revised.

1. The manuscript did not provide ethics approval.

Our ethical approval details are provided in the 'Study overview' section of the Methods lines 110-112. Nevertheless, we have now added an Ethical Approval statement to the footnotes section detailing the relevant ethical approvals and governance arrangements.

2. The study recruited participants from only two London hospitals. But London as a high-migrant-density city with relatively high TB incidence may have different healthcare accessibility compared to other UK regions. The discussion does not sufficiently address this unique characteristics of London. Recommendation: Expand the discussion to elaborate on the scope of generalisability, explicitly cautioning against uncritical application of findings to rural low-incidence areas in the UK or other national contexts.

We have expanded the discussion section (lines 418-420) to acknowledge that the study was conducted at two London hospitals, with relatively large migrant populations and high TB incidence. This may limit the generalisability of findings to other settings where the TB incidence, access to and awareness of services may differ.

3. The study mentioned telephone translation services for non-English speakers but did not specify how many participants used this service or discuss the potential impact of translation on data quality. In qualitative research, translation may alter semantic nuances. Recommendation: Supplement the methods with information on the number of participants requiring translation, the languages involved, and a discussion of how translation-related challenges were addressed (e.g., back-translation verification).

While access to telephone translation services was available to facilitate participation by non-English speakers, no participants required or used translation services during the study. We have added this clarification to the Methods section, lines 126-129.

4. The study found that knowledge had a mixed influence on treatment decisions but did not adequately explore how knowledge interacts with other domains (e.g., self-efficacy, perceived risk). Understanding these interactions could inform more effective patient education interventions. Recommendation: Enhance the discussion with an analysis of potential interaction effects between knowledge and other TDF domains, which may provide valuable insights for designing multifaceted interventions.

Thank you for your comment. We agree that the data show knowledge was an important influence on treatment decision-making. However, one of the core strengths of using the TDF is that it explores a holistic range of influences beyond capability factors alone. It is likely that an educational intervention would not be delivered in isolation for this reason, as motivational and opportunity factors also influence behaviour here. There were instances in the manuscript where interactions between capability, opportunity and motivational influences were drawn out during the narrative results, but we welcome your suggestion to enhance this further. As such we have made the knowledge-specific interactions more explicit in the Results and Discussion as follows:

- *Lines 196-199 "*Knowledge of side effects and treatment burden had a mixed influence on preventive treatment acceptance, in turn shaping participants' motivations, specifically their beliefs about their capabilities to manage treatment and side effects"
- *Lines 199-202 "*Participants' awareness of the broader context, including environmental risk factors, familiarity with the healthcare system, and the availability of NHS support in the event of TB becoming active, was itself influenced by their interactions with the physical environment around them and with the NHS, and also had a mixed impact on their treatment acceptance."
- *Lines 231-232 "*Contingent on participants' underlying understanding of TB and its treatment, captured under knowledge, participants…"
- *Lines 272-273 "*The accessibility of care for active TB shaped participants' knowledge of TB

and its treatment and reduced motivation for preventive treatment among some participants,"

- *Lines 335-340 "*Rather than acting in isolation, knowledge interacted with other domains. For instance, awareness that treatment does not provide lifetime immunity, or a perceived lack of robust evidence supporting the need for intervention, reduced the motivation to accept treatment. Similarly, knowledge of side effects and how they might be managed interacted with beliefs about capabilities, informing participants' confidence (self-efficacy) that they could tolerate and complete the regimen."
- *Lines 342-346 "*Partially influenced by their knowledge of TB and treatment, some participants adopted a preventive approach regardless of their future TB risk. For those participants, a high future TB risk tended to reinforce uptake, but a low risk was perceived as 'still a risk'. Other participants considered their risk of future TB too low to justify the treatment,.."

Reviewer: 2

Overall, this study is well conducted, well structured, and provides a clear description of the study context, methodology, and findings. One of the strengths of this study is that it captures perspectives from a diverse range of informants, including those who accepted TB preventive therapy, those who declined it, and those who remained undecided. This provides a relatively comprehensive and nuanced understanding of the factors shaping decision-making around TB preventive therapy.

Below are several suggestions that may further strengthen the manuscript:

5. Provide more context on the UK TB prevention and healthcare system. The UK, as a low-TB-burden country, provides an interesting and relatively unique context for this study. However, not all international readers may be familiar with how TB prevention and control programs are organized and delivered in the UK, including the financing and access mechanisms. I suggest adding a brief description of the UK TB prevention and control context in the Introduction, including how TB preventive therapy is offered, delivered, and financed. This would help international readers better understand the context in which the study was conducted and, importantly, allow them to draw broader lessons from the UK experience.

We have added a brief description of the UK context to the Introduction (lines 69-73), including the provision of TB preventive treatment through specialist NHS TB services and the fact that assessment, treatment and follow-up are provided free at the point of care. We also clarified that TB incidence varies across the UK with London having the highest rate. We hope this provides additional information with wider context for interpreting the study findings.

6. Reconsider the terminology "Prospective Cohort Qualitative Study." The term "Prospective Cohort Qualitative Study" feels somewhat unusual and may confuse readers. The term "cohort" is strongly associated with quantitative epidemiological research, in which participants are typically followed to estimate risks or associations using statistical methods. Therefore, I would suggest using a more conventional term, such as "Qualitative Longitudinal Research (QLR)" or "Prospective Qualitative Study," if this accurately reflects the actual study design.

We have revised the methods section in the manuscript to describe the work as a cross-sectional qualitative study using semi-structured interviews, which more accurately reflects the study design (see line 106).

7. Clarify the basis for describing the study as prospective. Related to the previous point, how do the authors justify describing this study as “prospective” if there were no repeated interviews or periodic observations of the participants? In qualitative prospective or longitudinal research, participants are generally followed over time, with qualitative data collected at multiple time points (e.g., through repeated in-depth interviews or observations). The purpose is not necessarily to estimate incidence or risk, but to explore how perceptions, experiences, emotions, social dynamics, or decisions evolve over time. The authors, therefore, need to clarify what makes the present study prospective. If participants were not followed longitudinally, it may be more appropriate to describe the design as an "exploratory qualitative study" rather than a prospective qualitative study.

Our inclusion of the word “Prospective” was initially to clarify that recruitment was done prospectively, following a pre-registered protocol (as opposed to a secondary analysis of existing data). Nevertheless, we have now amended this description as per the comment above (see line 106).

8. Reconsider the phrase “mixed influence on treatment acceptance.” The expression “mixed influence on treatment acceptance” feels somewhat inconsistent with the qualitative nature of this study, particularly since it does not seek to establish causal relationships between variables. I suggest using terminology that better reflects the interpretive nature of qualitative findings, for example, “dual considerations in treatment acceptance decisions,” “competing considerations in treatment decision-making,” or similar wording that captures how a particular factor may be perceived in different ways by participants.

We acknowledge the importance of using terminology that reflects the interpretive nature of qualitative research. We have retained the term "mixed influence" as it reflects factors that were perceived by participants as acting both as barriers and enablers to treatment acceptance. This aligns well to other research using behaviour change frameworks and models like TDF and COM-B, where barriers or enablers to a behaviour are often spoken about in terms of the influence they have on behaviour. In this instance this term is not there to suggest any level of quantifiable causality, but instead a qualitatively identified driver that either helps or inhibits behavioural performance. However, to improve clarity, we have revised the manuscript to explicitly define this category (see lines 158-159).

9. Further explain the absence of factors mapped to “Skills” and “Reinforcement.” The statement that “No factor was mapped into the domains of ‘Skills’ or ‘Reinforcement’ deserves further explanation and discussion in the Discussion section. This finding is potentially important, but it may be difficult for readers to interpret without first understanding the specific context of TB prevention in the UK and how TB preventive therapy is offered and managed within the healthcare system. At present, readers may be somewhat surprised by this finding. The authors could therefore discuss why these domains were not prominent in participants’ accounts and whether this may be related to the characteristics of TB preventive therapy delivery in a low-burden setting.

We have expanded the Discussion to further consider the absence of factors mapped to the ‘Skills’ and ‘Reinforcement’ domains. We note that this may reflect the nature of preventive treatment and

the way it is delivered in the UK, with participants placing greater emphasis on

knowledge, beliefs and motivations than on skills, rewards or incentives (see lines 373-379).

10. Strengthen the discussion through comparison with contrasting evidence and different TB-burden settings. The Discussion could be further strengthened by engaging with studies reporting findings that differ from, or even contradict, those observed in the present study. In addition, comparison with evidence from both high-TB-burden and low-TB-burden countries would be valuable. Such comparisons could help demonstrate whether the findings are context-specific or reflect broader patterns in TB preventive therapy decision-making. This would also provide a stronger basis for articulating the novelty and contribution of the present study to the existing literature.

We thank the reviewer for this suggestion. We have expanded the Discussion to compare our findings with evidence from both high- and low-burden settings. While several barriers identified in our study (e.g. concerns about side effects, stigma, and treatment burden) are consistent with previous qualitative studies, we now emphasise important contextual differences. In contrast to many studies from high-burden settings, where structural barriers, access to care and lack of knowledge are prominent drivers of non-uptake, participants in our UK setting generally had access to free specialist care. Decisions therefore centred more on perceptions of personal risk, uncertainty regarding the value of treatment, and treatment burden. We have also clarified that most prior data comes from higher burden countries, and existing data on factors affecting preventive treatment decisions in low incidence countries are lacking (e.g. see lines 380-391).

11. Elaborate further on the health-system and health-promotion implications. Finally, the implications of these findings for TB prevention and control in high-income, low-TB-burden settings deserve further elaboration. In particular, the authors could discuss how TB preventive therapy programs should be designed and communicated in such settings, and how health promotion and patient communication strategies may need to differ when TB is perceived as relatively uncommon. This discussion could be strengthened by integrating relevant findings from recent studies and current international guidelines and recommendations on TB prevention, particularly those addressing TB preventive therapy, patient engagement, and health-system approaches in low-burden settings.

We thank the reviewer for this suggestion. We have expanded the Discussion regarding the implications of our findings for TB prevention programmes in low-incidence settings (see lines 392-399). Specifically, we now highlight the importance of personalised risk communication and shared decision-making approaches that support patients in weighing perceived benefits and burdens of treatment. We additionally expand discussion of how shorter and better-tolerated regimens and new diagnostics may improve acceptability, and highlight the need for adequate support to address concerns about side effects and tolerability.
